# Immunological intervention in human diseases

**DOI:** 10.1186/1479-5876-5-59

**Published:** 2007-11-23

**Authors:** Hideki Ueno, Catherine M Hawrylowicz, Jacques Banchereau

**Affiliations:** 1 Baylor Institute for Immunology Research and Baylor Research Institute, 3434 Live Oak St, Dallas, TX, 75204, USA; 2 Allergy and Respiratory Medicine, King's College London, 5^th ^Floor Thomas Guy House, Guys Hospital, London, SE1 9RT, UK

## Abstract

A recent Keystone Symposium Meeting on "Immunological Intervention in Human Disease" was held in Big Sky, Montana on January, 6–11, 2007, organized by Jacques Banchereau, Federica Sallusto and Robert Coffman. It brought together basic scientists and clinicians from both academia and the pharmaceutical industry to discuss how the immune system is involved in the development of human diseases, including cancer, allergy, autoimmunity, and infectious diseases. We highlight advances in our understanding of the pathogenesis of immune-mediated diseases and future approaches in the immune therapeutic interventions. Considerable progress in the development of model systems and methodologies to monitor human immune responses will help to develop and to evaluate new immune-based therapies at pre-clinical and clinical studies.

## Introduction

Inappropriate immune responses cause a wealth of human diseases. Insufficient immune responses are associated with cancer or chronic infections. Conversely, excessive or undesirable immune responses are associated with allergy, autoimmune disease and the graft rejection in transplantation. Animal models, particularly the use of highly inbred and genetically-modified mouse strains, have been instrumental in advancing our understanding of the complex functions of the immune system in the past decades. Nevertheless, besides vaccinations, surprisingly few immunological treatments for immune-mediated pathologies are currently available. Notable exceptions include the use of monoclonal antibodies such as TNF antagonists (anti-TNF monoclonal antibody, or soluble TNF receptor) to treat rheumatoid arthritis and other chronic inflammatory diseases, and anti-CD20 monoclonal antibody currently used to treat B cell lymphomas and certain autoimmune diseases.

The meeting emphasized the importance of directly assessing the human immune responses, and that not all of what we learn in the mouse can be directly translated to humans. Importantly, one of the focuses of the meeting was to contribute to the education of a new generation of scientists and immunotherapists.

## Recent progress in human immunology

### Dendritic cells (DCs)

DCs play a key role in initiating and controlling the magnitude and the quality of adaptive immune responses [1]. Immature DCs decode and integrate such signals, and ferry this information to adaptive immune cells. The type of adaptive immune responses is highly dependent on the nature of the activating stimuli that DCs receive from the microenvironment. DCs are composed of subsets. There are two major human DC subsets, myeloid DCs (mDC) and plasmacytoid DCs (pDC). Human myeloid DC subset is further divided into two subsets, Langerhans cells and interstitial DCs, which carry distinct property to induce immune responses (J Banchereau; Baylor Institute for Immunology Research, TX). Langerhans cells are potent at priming antigen-specific naïve CD8^+ ^T cell responses, and inducing Th1 and Th2 cell responses, while interstitial DCs prime naïve B cells for development into IgM secreting plasma cells and promote CD4^+ ^T cell differentiation towards those specialized for help of antibody secretion from B cells (follicular helper T cells). It was proposed that Langerhans cells preferentially induce cellular immunity, while interstitial DCs preferentially drive humoral immunity (Banchereau), a concept that might impact effective design of vaccines, particularly in cancer and chronic infectious diseases. The second major DC subset, pDC is specialized for secreting type I IFN. Type I IFN secretion is impaired by the co-stimulation of unique receptors expressed by pDCs, such as ILT7, BDCA2, and NKp44 (Y-J Liu; MD Anderson, TX). All these pDC receptors are associated with FcεR1γ, which deliver strong inhibitory signals through an immunoreceptor tyrosine-based activation motif (ITAM) (Liu) [2].

In the steady state, DCs continuously capture antigens from dying cells, and present self-antigens to T cells, resulting in depletion or anergy of self-reactive T cells. Peripheral tolerance may be actively maintained by "tolerogenic" DCs, which induce the differentiation of T cells with regulatory/suppressor functions. DC10, defined as monocyte-derived DCs generated in the presence of IL-10, promote IL-10-secreting anergic CD4^+ ^T cells with suppressive functions (T regulatory type 1, Tr1 or IL-10-Treg). They express high levels of inhibitory immunoglobulin like transcript (ILT) receptors, particularly ILT4, and its ligand, HLA-G. The binding of HLA-G on T cells with ILT4 on DCs appears to be critical for the differentiation of induction of Tr1 (S Gregori; San Raffaele Telethon Institute, Italy). In mouse spleen, CD101 was proposed as a marker of tolerogenic DCs (J Bluestone; UCSF, CA), as CD101-expressing CD8α^- ^splenic DCs have capacity to convert naïve CD4^+ ^T cells into FoxP3^+ ^regulatory T cells in vitro.

### T cell subsets

The most recently described subset, Th17 cells, which preferentially secrete IL-17-family cytokines (IL-17F, IL-22, IL-26 and CCL20) [3], is involved in autoimmune diseases and acute inflammatory responses. Th17 cells also appear to play a critical role in the protection from infection and are essential for the recovery from *K. pneumoniae *infection in mice (J Kolls, Children's Hospital of Pittsburgh, PA).

Evidence of Th17 involvement in human diseases exists in multiple sclerosis (C Rohowsky-Kochan, New Jersey Medical School, NJ) and in psoriasis (R de Waal Malefyt; DNAX, CA). In mice, the development of Th17 and CD25^+ ^Foxp3^+ ^Treg in vitro is mutually exclusive. While the combination of TGF-β and IL-6 drives murine T cell differentiation towards Th17, TGF-β alone drives their differentiation towards FoxP3^+ ^Treg [4]. In humans, IL-1β and IL-6 represent key factors for Th17 differentiation in vitro, while IL-2, an essential growth factor for essentially all other T cell populations, antagonises Th17 development [5] (F Sallusto; Institute for Research in Biomedicine, Switzerland). Due to higher capacity to secrete these cytokines, monocytes are better than monocyte derived DCs in supporting the generation of Th17 (Sallusto). Other studies suggest IL-6 alone potently induces Th17 cells in vitro, IL-23 showing some additive effect (Rohowsky-Kochan). Several rounds of stimulation with IL-23 may be sufficient for the development of Th17 [3](de Waal Malefyt). In stark contrast to mice, TGF-β inhibited the differentiation of Th17 cells in humans (Sallusto and Rohowsky-Kochan). Human blood Th17 cells can be identified as a CD4^+ ^T cell subset expressing both CCR4 and CCR6 [5] (Sallusto).

The expression of chemokine receptors controls the migration of T cells, and allows identification of T cell subsets with distinct functions. For example, CXCR5^+ ^CD4^+ ^T cells represent a unique subset in the blood, which has potent capacity to prime naïve B cells (R Morita, Baylor Institute for Immunology Research, TX). CXCR3^+^CD8^+ ^T cells appear to be critical for the termination of primary immune responses [6]. CXCR3^+^CD8^+ ^T cells migrate to reactive, but not resting, lymph nodes (LN) in response to CXCL9, which is transiently secreted by inflammatory high endothelial venules (HEV). CTL that have migrated to the reactive LN then kill antigen-presenting DCs, resulting in inhibition of naïve CD4^+ ^and CD8^+ ^T cell priming (Sallusto).

### NK, NKT cells

Innate immune cells are involved in the first line of the response to microbial infections. However, their activity is tightly regulated by the adaptive immune system, probably to avoid excessive inflammation and host pathology. NKR-P1A (CD161) might be involved in the regulation of the activity of NK cells (L Lanier, UCSF, CA). LLT1, a type II disulfide bonded homodimer with a short cytoplasmic tail, was identified as the human ligand for NKR-PIA. LLT1 is expressed on pDCs activated with CpG and/or viral stimulation, and B cells stimulated with CpG or anti-CD40/IgM Abs. Stimulation through NKR-P1A inhibits NK cell cytotoxicity and IFNγ secretion. Thus, activated pDCs and B cells may regulate NK cell activity at the late stage of viral infections.

NK and NKT cells play an important role in immune surveillance against tumorigenic cells, and protective immunity to infectious agents. Human DCs exposed to α-galactosyl-ceramide (αGalCer), a synthetic NKT cell ligand, expand an NKT cell population, both in vitro and in vivo, that is capable of secreting large amounts of cytokines and killing target cells. This cognate interaction between NKT cells and DCs also leads to the potent activation of DCs, therefore NKT cells are proposed to act as an "adjuvant" for the induction of anti-tumor immunity in vivo (R Steinman, Rockefeller, NY). Expansion of NKT cells and antigen-specific T cells was observed after injection of αGalCer loaded mature DC into 5 advanced cancer patients [7] (Steinman).

Human NKT cells expressing invariant TCR recognize a wide repertoire of ligands including both foreign and self-glycolipids in the context of CD1d. Enormous variability in the frequency of NKT cells exists among healthy individuals and the genetic basis for this is ascribed to the SLAM superfamily at the level of NKT cell development in the thymus. An endogenous NKT cell ligand, iGb3, plays a critical role in NKT cell development (A Bendelac, Chicago, MI). A study of the crystal structure of a complex of human CD1d with αGalCer revealed the unique requirement of both A' and C' CD1d pocket-binding chain structure of αGalCer for the optimal stimulation of NKT cells (V Stronge, Oxford, UK). The discovery of novel ligands for NKT cells may lead to the identification of the optimal ligand for use as an "adjuvant" in vaccines for cancer or infectious diseases (A Bendelac, Chicago, MI).

## Novel discoveries and therapeutic approaches in human diseases

### Cancer

Active immunotherapy with cancer vaccine seeks to induce therapeutic anti-tumor immunity. Tumor-specific immune responses were successfully induced in patients by various vaccines, however, the overall rate of clinical response remains low. Thus, a deeper understanding of cancer pathogenesis is critical to improve the design of cancer vaccine.

Breast tumors attract DCs, which are modified to induce the differentiation of T cells towards inflammatory Th2 cells. The secreted IL-13 signals cancer cells as demonstrated by the presence of phosphorylated STAT-6 and contributes to tumor growth as demonstrated in humanized mice model described hereunder [8] (K Palucka, Baylor Institute for Immunology Research, TX). CXCR7, a chemokine receptor for CXCL11 and 12, may represent another potential target in cancer immunotherapy (B Summers, Mountain View, CA).

Immunity against cancer stem cells may be necessary to fully protect against cancer development and progression (M Dhodapkar, Rockefeller, NY). Cellular immunity against SOX2, a gene critical for self-renewal in embryonal stem cells, appears to block the malignant transformation of a benign monoclonal gammopathy of undetermined significance (MGUS), into multiple myeloma [9].

Cytostatic drugs such as anthracyclins promote anti-tumor immunity by inducing "immunogenic" tumor cell death. Two molecules are suggested to be key parameters in this process; calreticulin (CRT) [10], which is exposed on the cell surface of tumour cells, and high mobility group box 1 (HMGB-1), which is secreted by dying tumor cells. CRT facilitates tumor antigen uptake by DCs. HMGB-1 appears to associate with TLR4 and promote the uptake of tumor cells rather than the activation of DC. This observation is supported by evidence that a mutation in TLR4 (Asp 299 Gly), which blunts NFκB activation by HMGB-1 binding, correlates with poor survival in breast cancer [11](L Zitvogel, France).

Vaccines targeting idiotype protein expressed by lymphoma cells are known to induce immunological and durable clinical responses. Alternative strategies include the injection of autologous DCs into the tumor following adjuvant therapy such as radiotherapy or chemotherapy. Injecting CpG into the tumor after radiation therapy can further enhance anti-tumor immunity, an approach that does not require ex-vivo cell preparation (R Levy, Stanford, CA). A clinical trial for breast cancer using a MUC-1 peptide (100 mer) with adjuvant indicated the stabilization of the disease in 20% of vaccinated patient population (O Finn, Pittsburgh). Targeting Tregs might be an important strategy to increase anti-tumor immunity. Indeed, many patients with metastatic melanoma display melanoma antigen-specific Treg in the blood (H Ueno, Dallas, TX). These melanoma-specific Treg secrete large amounts of IL-10, and express FoxP3 and CTLA-4.

The adoptive transfer of tumor-antigen-specific T cells represents a passive form of cancer immunotherapy. Tumor antigen-specific CD8^+ ^T cell lines have been generated by transducing TCR α and β chains obtained from a T cell clone with high avidity for tumor peptide [12]. Introduction of cysteines into the constant region of the α and β chains facilitates preferential pairing of the transduced TCR chains, exclusion of endogenous TCR and the enhanced selection and expansion of tumor antigen-specific CD8^+ ^T cells (P Greenberg, Washington, WA).

### Infectious diseases

Novel vaccine strategies to HIV-mediated diseases were discussed. One strategy is to target DCs in vivo by an anti-DC-specific Ab conjugated to relevant antigens. In vitro, DCs incubated with HIV gag24 coupled to anti-DEC205 mAb induced efficient activation of gag-specific CD8^+ ^and CD4^+ ^T cells [13] (R Steinman). In vivo, a "prime-boost" vaccine strategy, priming with DNA vaccination followed by a boost with an adenoviral-vector based vaccine (encoding subtype B Gag-Pol-Nef fusion protein, and modified envelope (Env) constructs from subtypes A, B, and C), was shown to enhance anti-HIV cellular and humoral immunity (R Koup, NIH, MD). Induced HIV-specific T cells revealed more polyfunctional responses in HIV non-progressors than in progressors, suggesting that the quality of induced HIV-specific T cells have a great impact on the clinical outcome (R Koup). Another vaccine based on conjugates of the gag protein with a TLR7/8 agonist induced gag-specific CD4^+ ^and CD8^+ ^T cells capable of secreting multiple effector cytokines in nonhuman primates (R Seder, NIAID, MD). Vaccines with TLR7/8 ligand and gag without conjugation induced poor antigen-specific T cell immunity, and the secretion of fewer cytokines.

The capacity to generate effective T cell immune responses able to stop HIV replication in vivo represents an enormous clinical challenge, and T cell "exhaustion" during HIV infection may be an important contributing factor. PD-1 levels expressed on HIV-specific T cells correlate significantly with viral load during chronic HIV infection [14,15]. These PD-1^+ ^T cells represent "exhausted" T cells, which fail to respond to antigenic stimuli. Functional impairment could be reversed in vitro by PD-1/PD-L1 blockade (P-R Sekaly, Montreal, Canada).

### Inflammatory diseases/Autoimmune diseases

Psoriasis is a common autoimmune disease of the skin that affects about 2–3% of Western populations. pDCs accumulate in the inflamed skin at an early stage of the disease, and secrete type I IFN. LL-37, a peptide secreted from keratinocytes activates pDCs to secrete type I IFNs and may thus offer a novel therapeutic target [16](M Gilliet, MD Anderson, TX). LL-37 forms a complex with DNA, and prolongs DNA retention in early endosomes. This induces the potent activation of pDCs through TLR9, resulting in type I IFN secretion. mDCs at the psoriatic lesion secrete also IL-23, and thus are contributing to the development of Th17 cells which are involved in the pathogenesis of psoriasis (M Gilliet; R de Waal Malefyt, DNAX, CA). Th17 cells also promote the secretion of anti-microbial components from keratinocytes, such as β-defensins, which induce maturation of mDCs (R de Waal Malefyt). Another pathogenic T cell subset in psoriasis is VLA-1 (integrin α1β1)-expressing CD4^+ ^T cells that accumulate in the inflamed epidermis in psoriasis. Blocking VLA-1 prevents the migration of these cells into the tissue and inhibits the development of psoriasis, highlighting VLA-1 as a therapeutic target for psoriasis (F Nestle, King's College London, UK).

Systemic lupus erythematosus (SLE) is an autoimmune disease in which antibodies are formed against several nucleoproteins. SLE is associated with an increased production of Type I IFNs, most likely derived from pDCs following activation via TLR7 or TLR9. Novel approaches have been explored for the treatment of SLE using antagonists of TLR7/8/9 (G Lipford, Coley Pharmaceutical, MA) or TLR7/9 (R Coffman, Dynavax, CA). Lupus prone-mice receiving these antagonists generated less anti-dsDNA Ab than controls (G Lipford, R Coffman). Furthermore, an antagonist against TLR7/9 inhibited type I IFN secretion from pDCs activated with live influenza or herpes simplex virus as well as immune complexes (R Coffman).

In inflammatory bowel disease (IBD), pathogenic CD4^+ ^T cells express CCR9, a gut-homing chemokine receptor, which thus represents a novel therapeutic target. The administration of a small molecule antagonist of CCR9 reduces the disease severity in mouse models by blocking CCR9^+ ^T cell migration to the intestine (M Walters, ChemoCentryx).

High-dose intravenous γ-globulin (IVIG) therapy is a well-established treatment for a number of immune diseases, such as immunogenic thrombocytopenic purpura, and Kawasaki disease. Studies in animal models demonstrate that IVIG suppresses inflammatory macrophage functions by signalling through inhibitory Ig receptors (FcγRIIB). Sialylation of the Fc portion of IgG is critical for preferential binding to FcγRIIB, and the delivery of inhibitory signals [17](J Ravetch, Rockefeller, NY). A "sialic acid switch" model was proposed: There, steady state Ab is heavily sialylated, and thus anti-inflammatory, in order to maintain tolerance, while newly synthesised Ab is less sialylated, thus pro-inflammatory, and likely to promote the clearance of microbes (J Ravetch, Rockefeller, NY).

The use of antibodies targeting molecules expressed on pathogenic cells represents a recent success of immunotherapy. A B cell depleting therapy using anti-CD20 Ab (Rituximab) is beneficial for non-Hodgkin lymphoma as well as many B-cell mediated autoimmune diseases. The combination of anti-CD20 Ab and BR3 (BAFF receptor)-Fc chimeric protein, or anti-BR3 antibody induces a more prominent elimination of a wide repertoire of B cells including bone marrow plasma cells, and germinal center B cells (A Chan, Genentech, CA). A recent clinical trial in Type I diabetes used a non-mitogenic anti-CD3 Ab (hOKT3γ1, Teplizumab). Teplizumab led to stabilization of insulin production, preservation of islet function, and elimination of pathogenic T cells. Teplizumab also increased the frequency of distinct types of T regs, including IL-10^+^IFNγ-CD4^+^, Foxp3^+^CD4^+ ^and Foxp3^+^CD8^+ ^T cells (J Bluestone).

Nucleotide-binding oligomerization domain-Like Receptor (NLR) family of proteins are involved in the regulation of innate immunity and host defence against pathogens. (J Tschopp, Lausanne, Switzerland). Certain NLR family members promote the activation of pro-inflammatory caspases within multiprotein complexes, called inflammasomes. Cryopyrin/NALP3, the best-characterized NLR molecule, mediates caspase-1 activation upon binding of muropeptides, peptidoglycan fragments derived from the gram-positive and negative bacteria. NALP3 can also be activated by endogenous danger signals released from dying cells like uric acid [18]. Gout, which is induced by the deposition of monosodium urate (MSU) in joints, is also associated with the direct activation of NALP3 by MSU [18]. A constitutively active form of mutated NALP3/cryopyrin, which promotes IL-1β and IL-18 secretion by activating caspase-1, causes a familial auto inflammatory disease, Muckel Wells syndrome. Both diseases are rapidly treatable with IL-1 receptor antagonist (IL-1ra; Anakinra).

The nervous system also appears to control immunity, as shown by studies of the vagus nerve, whose stimulation prevents shock and tissue injury. Acetylcholine secreted from neurons directly acts on splenic macrophages, and suppresses the LPS-induced secretion of TNF and HMGB1. This suppression requires the α7 subunit of the acetylcholine receptor, and its agonist suppresses the cytokine secretion from macrophages (K Tracey, Feinstein Institute, NY). Thus, vagus nerve stimulation or stimulation with a α7 agonist could serve as a supportive general approach for the treatment of diseases associated with TNF, such as toxic shock and arthritis.

### Allergy, transplantation, and immunodeficiencies

Many elements of the allergic reaction, including the production of IgE antibodies and the recruitment and activation of eosinophils, depend on Type 2 cytokines, such as IL-4, IL-5, and IL-13. TSLP, which is secreted by epithelial cells, acts on DC resulting in the skewing of T cell responses towards an inflammatory Th2 phenotype, characterized by the secretion of high levels of Type 2 cytokines and TNF-α. IL-25, secreted from eosinophils, further promotes inflammatory Th2 development by TSLP-DCs. Th2 memory cells express the highest levels of IL-25R [19](Y-J Liu, Houston, TX).

Allergen immunotherapy represents an antigen specific treatment, but is associated with adverse effects in some patients due to whole allergen binding to IgE. A synthetic peptide vaccine containing the major T cell epitopes of *Fel d1*, the major allergen of cats, which reduces clinical responsiveness to cat allergen exposure in cat allergic subjects, induces IL-10-secreting specific T cells, and suppresses proliferation of allergen-specific Th2 cells (M Larche, Hamilton, Canada). Studies from an animal model and humans suggest intravenous injection of low or moderate doses of soluble peptide might induce peptide-specific immunological tolerance (M Larche).

In transplantation, recipient DC may act as key initiators of graft versus host disease (GVHD). Human LCs and dermal DCs undergo homeostatic proliferation in the skin [20], and these host-derived DCs induce alloreactive effector cells. Elimination of recipient DC prior to stem cell transplantation may be beneficial in preventing GVHD (M Merad, Mount Sinai, NY).

Life-threatening infections in children might be due to genetic/Mendelian factors. The herpes simplex encephalomyelitis (HSE) is caused in some cases by mutation of UNC-93B, a molecule controlling TLR3, 7, 8, 9 signalling [21](J-L Casanova, INSERM, France). This gene mutation abrogates type I IFN secretion as well as pro-inflammatory cytokine production upon TLR agonist ligation. Another genetic disorder responsible for HSE is a one-point mutation of TLR3, which results in impaired secretion of type-I IFNs in response to poly I:C stimulation [22]. These findings imply a novel therapeutic approach for the treatment of HSE with Type I IFN (J-L Casanova).

## Novel approaches for assessment of the human immune system

When considering the translation of basic research to medicine, ensuring both safety and efficacy are critical. Experiments performed in mice are not sufficient in this aspect, as clearly demonstrated by the recent tragedy in a phase I clinical trial with a super agonist anti-CD28 mAb [23]. In that trial, all six volunteers developed a very rapid systemic inflammatory response after the administration of the antibody, and required intensive organ support [23]. Thus, there is an urgent need for the development of tools and assays to assess the human immune system, and to predict its responses to novel therapeutic entities.

### Antigen-specific immunity

Active immunotherapy aims at inducing antigen-specific immune responses. Ultramulti-color flowcytometer has been used to assess the frequency (magnitude) and the type of cytokine secretion (quality) of antigen-specific CD4^+ ^and CD8^+ ^T cell responses in non-human primates vaccinated with HIV gag conjugated to TLR agonists (R Seder), or volunteers vaccinated against HIV using the "Prime-Boost" approach (R Koup). This methodology lead to the conclusion that a potent HIV vaccine should induce HIV antigen-specific CD4^+ ^and CD8^+ ^T cells with the capacity to secrete several cytokines. Assessment of antigen-specific T cell immunity also helps to understand immune (dys)regulation in diseases. CD4^+ ^T regs recognizing a broad range of melanoma antigens (NY-ESO-1, Survivin, TRP-1, and gp100) were identified by measuring IL-10 secretion in PBMCs obtained form patients with metastatic melanoma cultured with overlapping peptides (EPIMAX)(H Ueno).

There are fewer strategies to monitor antigen-specific B cells. Testing supernatants of PBMCs stimulated with CpG and IL-2 in vitro in multi-well plates for the presence of binding and/or neutralizing pathogen-specific antibodies. indicated that memory B cells secreting antigen-binding antibodies are more prevalent than those secreting neutralizing antibodies (A Lanzavecchia, Bellinzona, Switzerland).

### Antigen-non-specific immunity

Gene expressing profiling of follicular lymphoma biopsy samples showed a gene signature that negatively correlated with prognosis (L Staudt, NIH). This signature appears to be largely dependent on the presence of CD68^+ ^macrophages. In particular, CTGF (connective tissue growth factor) was found to be secreted by macrophages at tumor sites. Fibrosis at the tumor sites due to CTGF activation may be associated with the poor prognosis, possibly by hampering the anti-tumor immune responses (Staudt). In contrast to SLE which displays a typical type I IFN-mediated disease, microarray analysis of systemic onset juvenile idiopathic arthritis (SoJIA) revealed an important role of IL-1 in the development of the disease. Twelve genes were determined that allow the discrimination of SoJIA patients from patients with infectious diseases, SLE, or other autoimmune/inflammatory diseases [24]. This demonstrates that blood microarray can be used to identify diagnostic marker of disease (V Pascual, Dallas, TX) (Pascual).

### Humanized mice

Experimental therapy in humans is limited by ethical considerations. Mouse models in which the human immune system is reconstituted, termed humanized mice, are being developed to perform pre-clinical studies, such as analysis of the induction of human immune responses by vaccines in vivo. In one model, injection of human CD34^+ ^haematopoietic progenitor cells (HPC) into NOD-SCID β2m^-/- ^mice was followed by the adoptive transfer of autologous T cells. This model was used to demonstrate successful induction of flu-specific neutralizing IgM and IgG responses and priming of peptide-specific CD8^+ ^T cell responses following vaccination with antigen/peptide-loaded DCs (C I Yu and K Palucka, Dallas, TX). This model was further developed to study the interaction between DCs and cancer (epithelial tumors and melanoma). Implanting human breast tumor into these mice permitted to demonstrate the attraction of DCs at tumor site. Migrated DCs expressed OX40L, and promoted the development of inflammatory Th2 response. IL-13 blocking resulted in partial regression of the implanted tumors (Palucka).

A different humanized mouse model was generated by intrahepatic injection of CD34^+ ^HPCs into Rag2^-/-^γc^-/- ^mice (M Manz, Institute for Research in Biomedicine, Switzerland). The mice develop de novo human T, B, and DCs, have structured primary and secondary lymphoid organs, and mount antigen-specific humoral and cellular responses upon infection with EBV. Injection with CXCR5-tropic HIV-1 virus induces severe infection in the thymus and lymph nodes, resulting in systemic depletion of CD4^+ ^T cells as observed in humans. Infected cells include CD68-expressing macrophages as well as CD4^+ ^T cells (M Manz, Institute for Research in Biomedicine, Switzerland). Improved engraftment of human immune cells into Rag2^-/-^γc^-/- ^mice was demonstrated by pre-treatment with clodronate to kill phagocytic cells (H Spits, Genentech, CA). These mice develop human αβ and γδ T cells including Treg, as well as B cells, NK cells, and DC subsets. Generation of humanized mice with Rag^-/-^βc^-/- ^mice, where IL-7 signalling is intact, resulted in more peripheral LNs and T cells. One of the limitations of this model is the high turnover of human T cells in the mice, resulting in the low number of T cells. This could be partial overcome by transfecting BclxL into HPCs or co-injection of human fetal thymus and liver with HPCs. Further improvement of the humanized mouse models may allow us to study the full repertoire of human immune responses in such models (H Spits, Genentech, CA). Novel approaches to generate humanized antibodies with a "HuMAb" mice are under investigation (S Steven, Regeneron). Endogeneous murine antibody loci are replaced by human transgenes, providing a powerful tool to develop neutralizing high-affinity humanized antibody.

## Conclusion

Overall, it was an exciting and unique meeting focusing on translational research. Although translation of the knowledge from mice to humans is the common desire of scientists and the public, one must keep in mind that the immune systems of mice and humans differ in many aspects. Research with patients' samples is fundamental to understand human diseases and to create novel therapies. An infrastructure which promotes the study of human immune system and its involvement in diseases needs to be created by the collaboration with the translational scientists, the public, and the government. Only through this painful step, will we see a breakthrough in the treatment of human diseases.

## Authors' contributions

HU, CMH, and JB wrote the manuscript. All authors read and approved the final manuscript.
